# Soap in War Wounds

**Published:** 1918-11

**Authors:** 


**Soap in War Wounds.** Haycraft, Brit. M. J., Jan. 19, removes foreign bodies and dead tissue and cleanses with 25% soap solution. 91 of 98 cases thus treated and sutured primarily, healed by first intention. A few developed pyrexia and local reaction but returned to normal in a few days. He attributes a haemostatic action to the soap and, without claiming a positive antiseptic action, contends that it penetrates the crevices of wounds and removes debris mechanically. (Note: Some 30 years ago, a physician was discharged from a public position in Buffalo for having used soap suds in a case diagnosed as diphtheria. It may be that public sentiment owes a belated apology to him or his memory—we do not know which. At any rate, many isolated reports have indicated that, whether strictly speaking an antiseptic or not, soap may well supplant many antiseptics in numerous instances.)

